# Marine Sponge Natural Products with Anticancer Potential: An Updated Review

**DOI:** 10.3390/md15100310

**Published:** 2017-10-13

**Authors:** Cinzia Calcabrini, Elena Catanzaro, Anupam Bishayee, Eleonora Turrini, Carmela Fimognari

**Affiliations:** 1 Department for Life Quality Studies, Alma Mater Studiorum-University of Bologna, Corso d’Augusto 237, 47921 Rimini, Italy; cinzia.calcabrini@unibo.it (C.C.); elena.catanzaro2@unibo.it (E.C.); eleonora.turrini@unibo.it (E.T.); carmela.fimognari@unibo.it (C.F.); 2 Department of Pharmaceutical Sciences, College of Pharmacy, Larkin University, Miami, FL 33169, USA

**Keywords:** marine sponges, cancer, apoptosis, cell cycle, cancer chemoprevention, cancer chemotherapy

## Abstract

Despite the huge investment into research and the significant effort and advances made in the search for new anticancer drugs in recent decades, cancer cure and treatment continue to be a formidable challenge. Many sources, including plants, animals, and minerals, have been explored in the oncological field because of the possibility of identifying novel molecular therapeutics. Marine sponges are a prolific source of secondary metabolites, a number of which showed intriguing tumor chemopreventive and chemotherapeutic properties. Recently, Food and Drug Administration-approved drugs derived from marine sponges have been shown to reduce metastatic breast cancer, malignant lymphoma, and Hodgkin’s disease. The chemopreventive and potential anticancer activity of marine sponge-derived compounds could be explained by multiple cellular and molecular mechanisms, including DNA protection, cell-cycle modulation, apoptosis, and anti-inflammatory activities as well as their ability to chemosensitize cancer cells to traditional antiblastic chemotherapy. The present article aims to depict the multiple mechanisms involved in the chemopreventive and therapeutic effects of marine sponges and critically explore the limitations and challenges associated with the development of marine sponge-based anticancer strategy.

## 1. Introduction

Natural compounds represent a useful source of active molecules and play a noteworthy role in the drug discovery process. Inter alia, for several years, cancer therapy and prevention witnessed an exponential use of natural compounds as effective alternatives or supporting elements to traditional antitumor therapy. Nowadays, about 60% of all newly discovered anticancer drugs derive from natural compounds [1]. 

Cancer is a world burden. Malignant tumors still claim about 8.8 million victims per year and are the cause of 1 out of 6 deaths, globally [2]. Two major issues of current anticancer therapy are low efficacy and safety; as a result, the identification of new anticancer strategies, endowed with a better pharmaco-toxicological profile, to be used alone or in association with conventional chemotherapy, is essential. In this context, natural compounds could play a pivotal role since they are usually less toxic than traditional chemotherapy agents, they are effective, inexpensive and easily available [3]. In particular, natural compounds are often characterized by a better safety profile than traditional anticancer agents and the ability to inhibit cancer formation and development through the interaction with multiple cell signaling pathways [3]. This property makes it possible to affect multiple hallmarks of cancer at the same time, thus to effectively fight a complex disease, such as neoplasms. Furthermore, adapting Hippocrates’ precept “Primum non nocere” and following the World Health Organization’s (WHO) policy of prevention as a long-term strategy for the control of cancer, many natural compounds could represent potent chemopreventive agents.

If we ask ourselves what are the most renowned natural compounds currently used to cure major illnesses, we are sure that most readers would mention the cardiac glycoside digoxin or the antitumor taxanes or vinca alkaloids, all compounds obtained from the land. However, 70% of our planet is covered by water and marine biodiversity is unrivaled. This is the reason why medical research has focused on the marine world in the last six decades, starting to exploit its huge potential. Besides, modern resources and technological advances have made it possible to reach the unexplored sea depths, making marine biota accessible to researchers. Thus, in the 1950s Bergmann and Feeney [4] extracted for the first time an unusual nucleoside from the sponge *Tethya crypta*, which would later be renowned as the anticancer drug vidarabine [4]. Around 28,000 new compounds of marine origin were discovered [5]. Among all marine organisms, sponges, which belong to the phylum of *Porifera*, represent the richest source of natural marine compounds, contributing to 30% of all natural marine products identified so far [6]. This fact should not be surprising. Sponges are sessile invertebrates; they do not have an innate immune system or mechanic defense structures like shells or spines, therefore the only method they have of preserving themselves is to produce metabolites that act as a self-defense device and let them adapt to the environment [7]. Moreover, the biodiversity of sponges’ intraphylum translates into a chemical variety of the molecules produced. So far, particular nucleosides, sterols, alkaloids, peroxides, terpenes, fatty acids, amino acid derivatives and cyclic peptides have been discovered as active compounds and at least 60 of them have chemopreventive and/or anticancer potential [8]. These remarkable properties could be justified by the sponge’s ability to affect multiple cellular and molecular events, including DNA protection, cell cycle, apoptosis and inflammation as well as by their ability to chemosensitize cancer cells to traditional antiblastic chemotherapy.

The aim of this review is to illustrate and provide an update on the potential chemopreventive and antitumor effects of marine sponges, with particular focus on their ability to affect different pathways involved in cancer formation and development, and critically examine the limitations and challenges associated with the development of marine sponge-based anticancer strategy.

## 2. Proapoptotic and Anti-Inflammatory Effects

Apoptosis, or programmed cell death, is a physiological mechanism that occurs to preserve cellular homeostasis. It is essential for many biological activities, such as embryonic development, cell turnover, immune system maturation, and regulation of tissue homeostasis; accordingly, resistance to this process is itself a hallmark of cancer [9]. Furthermore, specific alterations in the apoptotic pathway are tumor distinctive, hence they may turn out to be a selective target for antitumor therapy [9].

Two main pathways can activate apoptosis: the mitochondrial or intrinsic pathway, and the receptor-mediated or extrinsic pathway. The intrinsic pathway refers to apoptotic cell death triggered by an enormous variety of intracellular damage or stress signals such as DNA damage, oxidative stress, endoplasmic reticulum (ER) stress, and cytokine withdrawal [10]. This pathway is regulated by the Bcl-2 family and involves mitochondrial outer membrane permeabilization (MOMP). Bcl-2 family counts pro- and anti-apoptotic proteins, and the balance between these elements decides the fate of the cell. Basically, when proapoptotic stress occurs, BH3-only proteins are recruited and activate Bax and Bak, which in turn trigger MOMP. As a result, cytochrome *c,* the second mitochondria-derived activator of caspases (SMAC) and other mitochondrial intermembrane space proteins are liberated into the cytosol. In this way, the apoptotic protease-activating factor 1 (APAF1) is able to reach cytochrome *c*, assembles the apoptosome, and activates caspase 9. Then, caspase 9 triggers the effector caspase-3 and caspase-7 that lead to apoptosis. At the same time, SMAC blocks the caspase inhibitor X-linked inhibitor of apoptosis protein (XIAP) leading to apoptosis. On their part, Bcl-2 homology (BH) 3-only proteins, and in particular Bid, can be activated by caspase-8 and lead directly to MOMP activation, thus representing the junction between the intrinsic and the extrinsic apoptotic pathway. Apoptosis induced by the extrinsic pathway occurs in response to extracellular stress signals like the pathogen- or damage-associated molecular patterns (PAMP or DAMP) or through the propagation of cytokines [10]. The extrinsic pathway requires the activation of specific receptors, such as Fas (CD95/TNFRSF6/Apo-1) or tumor necrosis factor (TNF)-family receptors, such as TNF-related apoptosis-inducing ligand (TRAIL) receptor (TRAILR). Upon binding with the designated ligands, a caspase cascade is triggered. At that point, adaptor proteins, such as Fas-associated death domain protein (FADD), make caspase-8 and caspase-10 dimerize, which in turn activate the effector caspase-3 and caspase-7, leading to apoptosis [10].

Intriguingly, inflammation, by now considered the seventh fully-fledged hallmark of cancer, has several converging pathways with apoptosis [9]. In fact, all TNF-receptor family members trigger both apoptosis and the activation of transcription factors leading to cytokine and chemokine production, but in a different chronological order [11]. While Fas receptor and TRAILR trigger first apoptosis and then inflammation, the TNF receptor 1 (TNFR1) first allows the translation of inflammatory genes and then apoptosis [11]. TNF-TNFR1 binding activates the nuclear factor kappa-light-chain-enhancer of activated B cells (NF-κB), c-Jun amino-terminal kinase (JNK), the mitogen-activated protein kinases (MAPKs), and p38 [11]. NF-κB is considered the most important link between cancer and inflammation. It is a transcription factor that regulates many biological processes, such as cytokine production, immunity modulation, cell growth, both cell survival and apoptosis, and tumorigenesis. Its constitutive activation has been described in several cancer types [12] and in all cancer phases (initiation, promotion and progression) [13]. NF-κB consists of heterodimers or homodimers of the NF-κB/Rel protein family, which mediate NF-κB dimerization, nuclear import, and specific DNA binding. Depending on the stimulus, NF-κB can be activated through different pathways. Microbial infections or inflammatory signals, such as TNFα and interleukin-1 (IL-1), act via IκB/IκB kinase (IKK). Briefly, in basal conditions NF-κB is coupled to its inhibitor IκB. When IKK is triggered, it phosphorylates IκB that in turn makes the p50-RELA-dimer free to reach the nucleus and allows the modulation of gene transcription. Otherwise, TNF cytokine family can induce the non-canonical pathway. This pathway requires the activation of IKKα by the NF-κB-inducing kinase (NIK). The formation of the complex NIK-IKKα-p100 (NF-κB subunit) leads to the phosphorylation of the p100 subunit and the nuclear translocation of p52-RELB dimer.

So far, more than 60 compounds obtained from sea sponges have shown their potential anticancer activity through the induction of apoptosis. For the majority of them, the proapoptotic mechanism is not completely elucidated and only their ability to induce one or more hallmarks of apoptosis has been demonstrated (Table 1). Some of them are at the same time involved in the inflammatory pathway. Selected compounds for which cellular and molecular mechanisms were explored in depth are presented and discussed in more detail in the text.

Renieramycin M is a tetrahydroisoquinoline extracted from the blue sponge *Xestospongia* sp. belonging to the same chemical family of ecteinascidin 743 (ET-743, Yondelis^TM^, or Trabectedin), the first marine anticancer agent approved by the European Union and by the Food and Drug Administration (FDA) for the treatment of advanced soft tissue sarcomas [74,75]. At nanomolar concentrations, it is cytotoxic for human colon, lung, prostate, brain, and breast cancer cells [73,76,77]. It conveys human non-small cell lung cancer H460 cells to p53-dependent apoptosis through the activation of the intrinsic pathway [72]. It is also able to stimulate anoikis. Anoikis is a particular type of apoptosis known as “detachment-induced apoptosis” and it is involved in the inhibition of cancer metastasization. Indeed, avoiding anoikis means avoiding apoptosis in cells that have lost adhesion to the extracellular matrix, allowing them to survive and develop a tumor [78]. Renieramycin M overcomes resistance to anoikis in H460 cells and in anoikis-resistant H460 cells [72,79]. Resistance to anoikis is associated with the up-regulation of phosphorylated extracellular signal-regulated kinase (p-ERK), phosphorylated ATP-dependent tyrosine kinase (p-AKT), Bcl-2, and Mcl-1. At subtoxic concentrations (0.5–2.5 μM for 24 h) [72], renieramycin M reduced all these protein levels [79]. Furthermore, renieramycin M decreased the expression of CD133, CD44 and ALDH1A1, which are markers of cancer stem cells [80].

Aaptamines are alkaloids extracted from different species of *Aaptos* sp. showing important potential anticancer activity. Dyshlovoy and coll. studied in particular three compounds (aaptamine, demethyl(oxy)aaptamine and isoaaptamine) and demonstrated their ability to trigger apoptosis in a monocytic leukemia cell line (THP-1). Among the three molecules, demethyl(oxy)aaptamine and isoaaptamine exhibited the strongest activity (Table 1). In a murine epidermal cell line (JB6 Cl41), all of them activated NF-κB and the transcription factor activator protein-1 (AP-1). Demethyl(oxy)aaptamine and isoaaptamine deregulated p53 transcriptional activity, while aaptamine did not affect it [14]. NF-κB and AP-1 partake in several biological processes, such as cell proliferation and migration, inflammation and apoptosis. AP-1 is a protein complex constituted by JUN, FOS, activating transcription factor (ATF), and musculoaponeurotic fibrosarcoma (MAF) family members that bind a shared DNA site, the AP-1-binding site [81]. NF-κB and AP-1 both have a dual role as tumor initiators and suppressors, depending on their composition, cell lines, microenvironment and stimuli [82]. In this respect, for example, a concurrent activation of these complexes is crucial to trigger apoptosis through the Fas ligand expression in leukemia cells (Jurkat) by DNA damaging agents [83]. Activation of AP-1 and NF-κB, and decrease in p53 levels are observed in the JB6 Cl41 response to cisplatin. For this reason, Dyshlovoy and coll. concluded that modulation of AP-1, NF-κB, and p53 is involved in aaptamine-mediated apoptosis [14].

Psammaplysene A (PsA) is a bromotyrosine derivative extracted from the marine sponge *Psammaplysilla* sp. Its potential anticancer activity has been explored in two endometrial cancer cell lines, Ishikawa and ECC-1. In both cell lines, PsA induced apoptosis and increased the expression of nuclear Forkhead box O1 (FOXO1) protein [60] FOXO1 is a transcription factor that plays an important role in the apoptotic process. Among others, FOXO1 activates the proapoptotic Bim protein or induces TRAIL-mediated apoptosis [84]. FOXO1 moves back and forth between nucleus and cytoplasm, creating a homeostatic equilibrium. Protein kinase B (Akt) can phosphorylate, inactivate and extrude FOXO1 from the nucleus, an event that inhibits apoptosis [85,86]. This event becomes pathologic when Akt is constitutively activated or when the tumor suppressor phosphatase and tensin homolog (PTEN) gene is mutated. Almost 50% of type I endometrial cancers are characterized by this mutation [87], and FOXO1 levels are significantly lower or absent in endometrial tumors [88,89]. To demonstrate that the induction of apoptosis and the increased expression of FOXO1 are related to each other, Berry et al. [60] evaluated the proapoptotic potential of PsA in silenced FOXO1 ECC-1 cells and in overexpressing FOXO-1 Ikasawa cells. Compared to the respective wild-type cells, PsA induced PARP cleavage to a lesser extent in the FOXO-1 silenced cell line, and to a greater extent in the overexpressing ones, demonstrating FOXO1 involvement in its modus operandi.

Stellettin B, a triterpene isolated from the marine sponge *Jaspis stellifera*, showed potential anticancer activity in various cell lines, including human glioblastoma (SF295), human non-small cell lung cancer (A549), and human chronic myeloid leukemia cells (K562 and KU812). In all four cell lines, Stellettin B induced an increase in the reactive oxygen species (ROS) levels that was linked to apoptosis. In leukemia cells, it triggered the mitochondrial pathway through upregulation of Bak and Bax protein levels, downregulation of Bcl-2, and induction of MOMP [46]. In SF295 and A549 cell lines, the pathway was not investigated [48].

Tang et al. [49] demonstrated that Stellettin B blocked the phosphorylation of Akt in SF295, with no effect on phospholipid kinase phosphatidylinositol 3-kinase (PI3K), its main activator. Thus, even if Stellettin B targets some Akt upstream proteins different from PI3K, the PI3K/Akt pathway is involved in its proapoptotic potential. Since their upstream effectors p38 and ERK were not modulated by Stellettin B, the involvement of the Ras/MAPK pathway was excluded. More recently, Chen et al. [47] and Wang et al. [48] confirmed Stellettin B’s ability to trigger apoptosis via Akt inhibition in A549, K562 and KU812 cells. However, as opposed to the evidence on glioblastoma cells, the authors showed the inhibition of the expression of p110, the catalytic subunit of PI3K, and the phosphorylation of phosphoinositide-dependent kinase-1 (PDK1), another Akt upstream protein [46,47]. An interesting experiment would be to evaluate the PDK1 levels in Stellettin B-treated SF295 cells. In the leukemic cell lines, Stellettin B inhibited Stat5 phosphorylation, a PI3K upstreaming protein, increasing the evidence that PI3K/Akt is involved in its cytotoxic mechanism [46]. In both leukemia and lung cancer cells, the expression of Akt downstream proteins were investigated after Stellettin B treatment, and a decrease in mTOR, a negative modulator of autophagy, was recorded, suggesting a role of this process in the potential antitumor activity of this sponge derivative [46,47].

The dibromotyrosine derivative (1′*R*,5′*S*,6′*S*)-2-(3′,5′-dibromo-1′,6′-dihydroxy-4′-oxocyclohex-2′-enyl) acetonitrile (DT), isolated from the sponge *Pseudoceratina* sp., showed proapoptotic activity on leukemia K562 cells. Relying on ROS production, it activated the intrinsic pathway, as demonstrated by the activation of caspase-9, and induced the disruption of MOMP. In addition, different mechanisms intertwine in DT activity. Certainly, oxidative stress plays a pivotal role in its potential antitumor activity. Indeed, oxidative stress seems to drive the modulation of the other two pathways involved in its cytotoxicity, i.e., PI3K/Akt and IKK/NF-κB [61].

Su et al. [61] showed that, unlike PsA and Stellettin B, DT induced apoptosis through the inhibition of FOXO genes and the activation of PI3K/Akt. DT represses PTEN activation and stimulates Akt phosphorylation that would lead to FOXO deactivation.

The IKK/NF-κB pathway is involved in different cellular events, such as inflammation or cell survival [90] and it has been demonstrated that ROS-mediated apoptosis is linked to the repression of this pathway [91,92]. DT reduced IKK/NF-κB downstream protein expression via ROS stimulation, suggesting its implication in DT-induced K562 apoptosis [61].

Another cellular mechanism involved in sponge-mediated apoptosis is ER-stress. ER-stress is triggered when an imbalance between protein folding capacity and functional demand occurs. The most common pathway involved in ER homeostasis restoration involves the unfolded protein response (UPR). UPR compensates for the accumulation of misfolded or unfolded proteins, but if stress is prolonged or the adaptive response is unsuccessful, apoptosis ensues. Protein kinase RNA (PKR)-like 67 ER kinase (PERK) is one of the ER transmembrane receptors that govern UPR. Among other kinases, both PERK and PKR can phosphorylate and inhibit eukaryotic translation initiation factor 2 subunit 1 (eIF2α). When eIF2α is phosphorylated, it is unable to efficiently initiate translation, leading to the inhibition of global protein synthesis and to apoptosis mediated by different pathways [93].

Candidaspongiolide (CAN) causes apoptosis in human U251 glioma and HCT116 colon carcinoma cells in a singular way. It triggers caspase-12 and, to a lesser extent, caspase-3 activation. Caspase-12 is involved in ER stress–induced apoptosis in rodents [94], while its role in humans is not clear yet [95,96]. Nonetheless, ER-stress was demonstrated to play a crucial role in CAN-induced apoptosis in the human cancer cells tested. In fact, CAN phosphorylates eIF2α through PKR activation [20]. Of note, UPR was not involved in CAN-mediated ER stress [20]. Since eIF2α phosphorylation is essential for CAN-mediated apoptosis and other UPR effectors, such as C/EBP homologous protein (CHOP) were not modulated, Trisciuoglio and coll. presumed that the PKR/eIF2α pathway activates NF-κB or the FADD/caspase-8 and Apaf-1/caspase-9 pathways, leading to apoptosis [20]. 

The methanol and hexane extracts of the marine sponge *Geodia cydonium* showed potential anticancer activity on different rabbit colon carcinoma and myosarcoma cell lines, even if the mechanism of action has not been investigated [97]. On the contrary, the methanol extract did not exert any cytotoxic or proapoptotic effect on breast cancer cells (MCF-7) [98]. However, in the same cell line, the extract exhibited an anti-inflammatory activity [98]. It decreased vascular endothelial growth factor (VEGF) levels and other proinflammatory cytokines such as interferon-α (IFN-α) and TNF-α, mutually interconnected with NF-κB. Moreover, it directly downregulated the gene expression of two NF-κB subunits, NF-κB1 and c-Rel [98]. All this information suggests that *Geodia cydonium* is endowed with an anti-inflammatory potential mediated by NF-κB inactivation.

Heteronemin is a sesterterpene isolated from the sponge *Hyrtios* sp. that modulates the transcriptional level of several pathways related to cell survival, such as cell cycle and apoptosis, and inflammation. Heteronemin triggers both the intrinsic and the extrinsic apoptotic pathways in chronic myeloid leukemia K562 and human renal cell carcinoma A498 cells [39,42] and negatively modulates NF-κB at subtoxic concentrations in K562 [39]. Precisely, it inhibited the PI3K/AKT pathway and the MAPK family member ERK in A498 cells [39,42]. In K652 cells, heteronemin targets NF-κB upstream protein MAPK, sensitized K652 cells to TNF-α-mediated apoptosis, and inhibited the phosphorylation of p38, another MAPK protein that turned out to be crucial for heteronemin-mediated apoptosis, and c-Jun NH2-terminal kinase (JNK) [42]. JNK belongs to the MAPK family and could trigger apoptosis or autophagy. In the heteronemin-treated A498 cells, it led to autophagy [42]. One interesting fact is that the inhibition of autophagy significantly increased heteronemin-induced apoptosis [42]. Thus, the combination of heteronemin with autophagy inhibitors could be an intriguing therapeutic strategy.

The precise mechanism of the potential anticancer activity of heteronemin has not been completely elucidated and, since MAPK is involved in both anti-inflammatory and proapoptotic pathways, further studies are required to understand the role of its modulation in the proapoptotic and anti-inflammatory potential of heteronemin [39].

Dideoxypetrosynol A is a polyacetylene isolated from *Petrosia* sp. known for its proapoptotic activity on human skin melanoma SK-MEL-2 and human leukemia U937 cells mediated by the mitochondrial pathway [58,99]. In U937 cells, it exhibited very interesting anti-inflammatory properties through the inhibition of the arachidonic acid (AC) pathway. Cyclooxygenase-1 (COX-1) and cyclooxygenase-2 (COX-2) are two crucial enzymes in AC metabolism. COX-1 is the constitutive form, while COX-2 is inducible [99]. These two enzymes catalyze the conversion of AC to the inflammatory mediator prostaglandins (PG). An over-induction of COX-2 could bring to an overproduction of PGE_2_ and facilitates the proliferation of neoplastic cells [100,101]. COX-2 up-regulation has been described in several kinds of cancer and is involved in carcinogenesis, metastatic potential, and angiogenesis [102]. Dideoxypetrosynol A markedly downregulated COX-2 mRNA and protein expression in U937 cells, while it did not affect COX-1 levels. It also induced a dose-dependent decrease in PGE_2_ amount [99].

Other crucial promoters and mediators of inflammation are the proinflammatory cytokines, such as the interleukin family. For instance, interleukin 8 (IL-8) is involved in many inflammatory disorders, from arthritis to cancer. It activates neutrophiles and, at the same time, has chemo-attractant properties. It promotes angiogenesis in physiological situations, such as wound healing, but in case of existent tumors this property could be linked to cancer ability to create new blood vessels, activate survival-signaling pathways, and promote metastasis [103]. Thus, the inhibition of IL-8 can provide a strategy to contrast cancer-related inflammation. Theopederins K and L from the marine sponge *Discodermia* sp. and mycalamide A from *Mycala* sp. are non-specific protein synthesis inhibitors able to inhibit IL-8 secretion in different pancreatic cancer cell lines [104]. Their ability to block IL-8 deserves more in-depth study to fully understand their impact on the tumor process. 

## 3. Antiproliferative Effects

Cancer cells are characterized by sustaining proliferative signaling that could result from the insensitivity to proliferation-inhibitory signals arising from the stroma or specific gene expression patterns [105]. Many marine compounds display antiproliferative activity targeting the modulators of the cell-cycle progression. A list of marine spongean compounds with antiproliferative activity is presented in Table 2. Selected compounds for which cellular and molecular mechanisms were explored in depth are presented and discussed in more detail in the text.

Aragusterol A is a steroid isolated from *Xestospongia* sp. that exhibits antiproliferative activity on many human cancer cell lines (breast, ovarian and cervical cancer, lung, oral epidermoid, gastric, colon cancer and leukemia) [111]. It inhibits protein and nucleic acid synthesis and induces cell-cycle arrest at late G1 phase in human non-small cell lung cancer. The cell-cycle block was dependent on the reduced expression of CDKs and cyclins involved in the G1-S transition, such as CDK2, CDK4, cyclin D1, A, and E. In particular, the decrease in the complex cyclin E-CDK2 activity led to a reduced pRb phosphorylation and, consequently, to an antiproliferative effect [135].

(19*Z*)-Halichondramide is a macrolide produced by *Chondrosia corticata*. It showed antiproliferative activity against non-small cell lung cancer cells (A549) through the block in the G2/M phase. The arrest in the G2/M phase was related to an increase in p53 and GADD45 (growth arrest and DNA damage), and to the downregulation of their downstream target genes including cyclins B1 and A, CDC2 and its phosphatase activator CDC25C [120]. Additionally, (19*Z*)-Halichondramide suppressed the constitutively activated Akt/mTOR pathway, responsible for cell proliferation and survival. In particular, this natural compound induced the downregulation of mTOR and in the same way reduced the expression of the mTOR precursor Akt, its survival effectors p70 S6 kinase and the eukaryotic initiation factor 4E (eIF4E)-binding protein 1 (4EBP1). Furthermore, a downregulation was noticed of the AMP-activated protein kinases (AMPKs), ERK, and p38, which regulate cell proliferation [120].

Smenospongine is a sesquiterpene founded in *Dactylospongia elegans* [23]. It has antiproliferative effects in a human chronic myelogenous leukemia cell line (K562) through the cell-cycle arrest in G0/G1 phase mediated by p21 increase and p57 decrease [134]. Concurrently with the G0/G1 block, an increase in peroxidase activity, a marker of hemoglobin production, was noticed. K562 differentiation was also confirmed by the increase in glycophorine A, a surface sialoglycoprotein expressed during erythroid differentiation [136], and the inhibition of Crkl phosphorylation by Bcr–Abl tyrosine kinase, a marker of chronic myelogenous leukemia pathogenesis [134,137]. A more recent study showed the antiproliferative effect of smenospongine also in other leukemia cells (HL60 and U937), characterized by a G0/G1 arrest. Furthermore, the study reported the involvement of the p21-pRb pathway in the smenospongine-mediated G0/G1 arrest, but no effect of the marine compound on p21 promoter was recorded, leaving how smenospongine increases p21 unclear [23]. Likewise, the spongean sesteterpene PHC-1–PHC-7 induced K562 differentiation as demonstrated by G0/G1 arrest, and increased hemoglobin production, glycophorine A expression, and enucleation of cells [129].

Another compound inducing K562 differentiation is Crambescidin 800, an alkaloid isolated from *Monanchora ungiculata*. Like smenospongine, crambescidin 800 increased hemoglobin peroxidase activity and p21 expression; no alterations in p27 expression were noticed. Unlike smenospongine, it caused an accumulation of cells in the S-phase [114]. Furthermore, morphological changes with development of neurite formation were observed in murine neuroblastoma cells treated with crambescidin 800 [114]. A G0/G1 block was recorded in a human hepatocellular carcinoma cell line treated with crambescidin 800 or, more powerfully, with crambescidin 830 and 816. The cell-cycle block was accompanied by the downregulation of gene expression of CDK1 and 2, cyclins A and D, and by the upregulation of some cyclin-dependent kinase inhibitors (CDKN2A, 2D and 1A) at mRNA levels. Crambescidins also activated p53-downstream target genes, such as GADD45 and 14-3-3-σ, which in turn inactive CDK1 and lead to cell-cycle arrest. At the same time, PUMA, another p53-target gene, was activated, leading to apoptosis via the intrinsic pathway (see also Table 1) [22].

The cell-cycle arrest could be the consequence of the alterations in microtubule-tubulin kinetics since they play a pivotal role in mitosis and cell division: the microtubule network reorganizes, in a highly coordinated manner, into a mitotic spindle that attaches and separates chromosomes. Therefore, over the years, several anticancer agents targeting microtubule have been developed. These compounds bind tubulin and induce microtubule stabilization or destabilization, which leads to disruption of the mitotic spindle, cell-cycle block in M phase and, eventually, cell death [138]. The best-known antitubulin drugs are taxoids and vinca alkaloids. Microtubules are made up of 13 parallel protofilaments arranged in a circle. The polymerization occurs by the addition of α,β-tubulin heterodimers and GTP hydrolysis at the plus ends; the depolymerization process, instead, occurs with the release of GDP at the minus ends. Stabilizing agents, such as vinca alkaloids, induce assembly of heterodimers by increasing lateral protofilament interactions. Destabilizing agents, such as taxanes, reduce polymerization by inducing an altered conformation [139].

Marine sponge-derived compounds, such as avarols, arenastatins, halichondrin, jaspolide, milnamide, and spongistatin are microtubules-destabilizing agents; instead, dactylolides, dictyostatin, discodermolide, hemiasterlin, laulimalide, peloruside A, and zampanolide act as stabilizing microtubules [140]. However, clinical studies for many of them (avarols, arenastatins, spongistatins, dactylolides, dictyostatins, laulimalides, and zampanolides) are lacking because of their low availability in nature or long synthesis processes, rapid inactivation, or toxicity [141].

Laulimalide and the less potent isolaulimalide are poor substrates of the P-glycoprotein (Pgp) efflux pump, as demonstrated in human ovarian cancer cells overexpressing Pgp and vinblastine-resistant [18]. Likewise, discodermolide is effective in human colon and ovarian carcinoma cell lines overexpressing Pgp and resistant to paclitaxel [142]. Peloruside A is also not efficiently extruded by Pgp since its cytotoxic activity was similar in multidrug resistance (MDR)-overexpressing Chinese hamster ovary cells and Pgp-overexpressing human ovarian carcinoma cells resistant to paclitaxel, compared to parental cell lines [143].

Peloruside A, isolated from *Mycale hentscheli*, is a microtubule stabilizer that binds β-tubulin at the laulimalide/peloruside domain, promotes G2/M arrest, and induces the formation of micronuclei, multiple bundles of microtubules and asters in mitosis, as shown in lung adenocarcinoma cells [127,143]. The patent for peloruside A belongs to the University of Victoria [144]. A study by the same University recently demonstrated that peloruside A forms a hydrogen bond with aspartic acid 297 of human β-tubulin. This evidence suggests a unique mechanism for microtubule stabilization of peloruside A [145]. 

A clinically important observation is that peloruside A proved to be more effective in reducing tumor volume compared to docetaxel and paclitaxel in two lines of human non-small cell lung cancer implanted in athymic *nu*/*nu* mice: 74–99% of tumor growth inhibition depending on the cell line implanted against 40–50% induced by taxanes. Of note, peloruside A was better tolerated than taxanes. However, the combination therapy of peloruside A plus docetaxel or paclitaxel resulted in high toxicity and mortality [146]. In a further study performed on Pgp–overexpressing ovarian xenografted-bearing mice, peloruside A was less effective in tumor growth inhibition than doxorubicin and paclitaxel; it was, however, better tolerated, with 14% of animal death against 43% recorded for the reference drugs [146]. Currently, clinical studies are on standby due to the limited quantities of peluroside A obtained from natural matrix. To overcome this problem, much effort is therefore being made towards upscaling its synthesis [147]. Another issue to be considered is the resistance to peloruside A recorded in in vitro experimental settings. Resistance seems to be linked to mutations in β-tubulin and downregulation of vimentin [148,149]. Moreover, a point mutation at D297 residue resulted in resistance to peloruside A [145].

PM060184, isolated from the marine sponge *Lithoplocamia lithistoides*, is a microtubule-destabilizing agent that inhibits the addition of further tubulin subunits at the plus ends of microtubules or creates a complex with α/β-tubulin dimers [150]. Through the binding with the maytansine site, it impedes the formation of longitudinal tubulin–tubulin interactions [150], leading to disruption of microtubules and cell-cycle arrest in G2/M phase [131]. It exhibited high antiproliferative activity in 24 cancer cell lines at very low concentrations [average IC_50_ (the drug concentration that inhibits cell proliferation by 50% compared with untreated cells) = 0.7 nM] [131]. The antiproliferative activity was also confirmed in six xenograft models of human cancer. The administration of 16 mg/kg of PM060184 on days 0, 7 and 14 did not induce systemic or local toxicity and determined an overall tumor regression in four models [132]. Moreover, the antiproliferative activity was maintained in in vitro and in vivo models with Pgp-mediated chemoresistance [131].

## 4. Chemosensitizing Properties

The resistance to anticancer drugs is still one of the major problems that determines the failure of antiblastic chemotherapy. Some types of tumor have an innate ability to resist antineoplastic drugs, but many others develop chemoresistance during or after therapy, resulting in refractory cancers. When resistance against drugs with different mechanisms or structures occurs, it is defined as MDR. One of the most frequent mechanisms of resistance is due to an increased efflux of drugs out of the cells, mediated by the ATP-binding cassette (ABC) transporters. Forty-nine members belong to this protein family and the three most common transporters related to MDR are: Pgp (also known as multidrug resistance protein 1, MDR1), MDR-associated protein 1 (MRP1), and breast cancer resistance protein (BCRP) [151]. Normally present in almost all tissues at low levels, these efflux pumps are expressed at higher levels in epithelial cells with secretory functions (intestine, kidney, liver, placenta, and blood-brain barrier). Their overexpression in many types of cancer leads to intrinsic drug resistance against anticancer drugs, such as vinca alkaloids, anthracyclines, taxanes, camptothecines, and epipodophyllotoxins [152]. However, these proteins are also highly inducible in cancer tissues. For example, a single doxorubicin perfusion increased 3–15-fold MDR1 gene expression in less than 1 h in biopsies of sarcoma pulmonary metastases, whereas no alteration was noticed in normal lung tissues of the same patients [153]. All three proteins have similar substrate specificity and promote the elimination of hydrophobic compounds. Furthermore, MRP1 and BCRP extrude metabolic conjugates, differently from Pgp [154]. To counteract MDR, three classes of ABC transporter inhibitors have been synthesized [155]. In vitro, inhibitors of these efflux pumps are able to sensitize resistant cancer cells; however, they proved less efficient in in vivo models. Research is therefore still moving towards the identification of new compounds acting as chemosensitizing agents and to be administered in association with antiblastic therapy.

In the following sections, we present some examples of associations of marine spongean compounds and anticancer drugs, focusing on the mechanisms of the interaction. Experimental details and pharmacological effects of these compounds are summarized in Table 3.

Lamellarin O is an alkaloid isolated from the Australian marine sponge *Ianthella* sp. It was studied as an inhibitor of ABC transporters known to induce MDR [163]. Cytotoxicity, accumulation and efflux studies were performed on human cancer cell lines (both wild-type or overexpressing one of the ABC transporters). Lamellarin O inhibited (1) Pgp in human Pgp-overexpressing colon cancer cells reversing P-gp mediated doxorubicin resistance; (2) MRP1 in human MRP1-overexpressing ovarian carcinoma cells; and (3) BCRP in human mitoxantrone-resistant non-small cell lung cancer cell lines increasing intracellular mitoxantrone [163]. Western blot and in silico docking analyses identified the BCRP inhibitory pharmacophore of lamellarin O [163].

Aragusterol A is a steroid isolated from *Xestospongia* sp. It was found to inhibit the growth of cisplatin- and doxorubicin-resistant cancer cells. For example, the cisplatin IC_50_ in cisplatin-resistant non-small lung cancer cells was 30.3 μM (versus 0.18 μM in their parental drug-sensitive cells); the IC_50_ of aragusterol A was 0.18 μM (versus 0.42 μM in the drug-sensitive cells). Instead, a partial cross-resistance was found in two human doxorubicin-resistant cell lines. Indeed, the doxorubicin IC_50_ in doxorubicin-resistant leukemia cells was 5.05 μM (versus 0.10 μM in the drug-sensitive cells); the IC_50_ of aragusterol A was 0.73 μM (versus 0.12 μM in their parental cells). Even if the resistance to doxorubicin is usually mediated by Pgp, the possible interaction between the marine steroid and Pgp was not investigated [111].

Agosterol A, a spongean sterol isolated from *Spongia* sp., was able to reverse multidrug chemoresistance to vincristine, colchicine, doxorubicin, and etoposide in human epidermoid carcinoma cells overexpressing Pgp or MRP1 [164,165]. The natural compound increased the intracellular accumulation of vincristine and reduced its efflux, restoring intracellular drug concentrations similar to those in the parental drug-sensitive cell line. The ability of agosterol A to inhibit efflux transporters was due to its direct interaction with both Pgp and MRP1 [164]. Moreover, a study revealed its ability to competitively inhibit the transport of amphipathic substrates by MPR1. Agosterol A was also able to decrease glutathione intracellular levels, lowering the formation of drug-glutathione conjugates, which are substrates of MRP1. The glutathione depletion was independent of its activity on MRP1, since a similar decrease was noticed also in the non-MRP1-overexpressing cells [165].

The sipholane triterpenoids (sipholenol A, sipholenone E, sipholenol L, and siphonellinol D), isolated from the sponge *Callyspongia siphonella,* were studied as Pgp inhibitors [166,167]. They were able to enhance the cytotoxicity of paclitaxel, colchicine and vinblastine in drug-resistant human epidermoid carcinoma cells overexpressing Pgp. No altered IC_50_ was observed in the original clone. The triterpenoids were specific inhibitors for Pgp. In fact, in the same cell lines, they had no effect on the IC_50_ of cisplatin, which is not a substrate of this glycoprotein. Furthermore, they did not modify the cytotoxic profile of the above reported anticancer drugs when administered to cell lines knockout for Pgp or expressing other types of resistance proteins (i.e., MDR1, MDR7, or BCRP) [166,167]. The sipholanes’ ability to reverse chemoresistance and increase the intracellular accumulation of drugs is due to a decreased efflux activity of Pgp by a direct interaction with the substrate binding-site of Pgp. The compounds lack effects on Pgp expression [166,167].

Panicein A hydroquinone, isolated from the Mediterranean sponge *Haliclona (Soestella) mucosa*, was found to increase the cytotoxic and proapoptotic activity of doxorubicin in two human melanoma cell lines (MEWO metastatic cells and malignant melanoma A375 cells) [161]. Both cell lines express the protein Patched, which is the receptor for the Hedgehog signaling pathway. This pathway regulates many processes during embryonic development. The aberrant activation of this pathway is linked to tumorigenesis and metastatization as well as resistance to antiblastic drugs [168]. Patched is also a multidrug efflux pump that extrudes many drugs out of cells. The synergistic effect observed for the doxorubicin plus panicein A hydroquinone association was due to a reduced doxorubicin efflux by Patched receptor [169]. The analysis performed on a structural model of Patched identified a panicein-binding site near to that for doxorubicin, suggesting a possible interference of the marine compound with Patched drug efflux activity [161]. Since Patched is expressed prevalently in cancerous cells in adults, its inhibitors could have a higher selectivity than compounds acting on ABC transporters, which are expressed ubiquitously. Furthermore, the cytotoxic effect against cancerous cells overexpressing a gene target of the Hedgehog pathway was not observed on non-transformed cells [170].

Fascaplysin, an alkaloid isolated from the *Fascaplysinopsis bergquist* sp. [171], induces ROS generation, cell-cycle arrest, and apoptosis [160]. It showed a synergistic cytotoxicity with the topoisomerase I inhibitors camptothecin, 10-hydroxycamptothecin and topotecan on chemoresistant NCI-H417 small-cell lung cancer. The mechanism of action is still unclear. However, fascaplysin could interfere with the topoisomerase I activity since no interaction or minor antagonism was found with other classes of antineoplastic drugs, such as DNA crosslinkers (cisplatin, carboplatin, oxaliplatin and mitomycin), topoisomerase II inhibitors (etoposide), DNA intercalating agents (doxorubicin), inhibitors of microtubules (vinblastine, and docetaxel), nucleoside analogs (gemcitabine and cytarabin) [160].

Manzamine A is an alkaloid found in the sponges *Haliclona* sp., *Pachypellina* sp., *Pellina* sp. and *Xestospongia* sp. It is endowed with various pharmacological properties, such as potential anticancer, antimicrobial, and anti-inflammatory activities [172]. This marine compound increased the sensitivity to TRAIL-apoptosis inducers in human pancreatic adenocarcinoma cells [173].

A useful strategy to improve the clinical application of an anticancer drug might be to widen its therapeutic window. Adverse effects are the limiting factors of many anticancer therapies, such as nephrotoxicity and ototoxicity for cisplatin [174,175], cardiotoxicity for doxorubicin [176] or pulmonary toxicity for bleomycin [177]. Natural compounds with selective cytoprotective abilities in non-transformed cells could therefore allow the use of higher concentrations of anticancer drugs.

Aaptamine and aeroplysinin-1 are two alkaloids derived from *Aaptos suberitoides* [178] and *Aplysina aerophoba* [179], which were studied as cytoprotective agents in rat tubular kidney cells and rat glomerular endothelial cells. Reduced cytotoxicity and genotoxicity were observed with 1–2 μM of alkaloids only in cisplatin-treated cells; instead, no protection was noticed against oxaliplatin or doxorubicin [180]. Aaptamine showed greater cytoprotective effects compared to aeroplysinin-1 in normal cells, and was more selective than aeroplysinin-1 in human bladder carcinoma cells. Aaptamine did not alter cisplatin or oxaliplatin cytotoxicity in transformed cells, whereas aeroplysinin-1 induced a slight resistance to platinum compounds [180]. However, the cytoprotective mechanism of these spongean alkaloids is still unclear. They seem not to influence cisplatin influx/efflux because cisplatin-DNA adducts formation weren’t reduced. Of note, they activated the checkpoint kinase-1 and induced a reduction of Bax, suggesting that these compounds modulate the DNA-damage response mechanisms [180].

The inhibition of G2 phase DNA-damage checkpoint (Chk1 and 2) could be a useful strategy to sensitize cancer cells to DNA-damaging anticancer drugs. DNA damage actives the phosphoinositide kinases ataxia-telangectasia muted (ATM) and ATM- and Rad3-related (ATR) , which transmit the signals to Chk1 and Chk2. Chk1 and Chk2 in turn phosphorylate Wee1 kinase and Cdc25 phosphatases. Both actions lead to the inactivation of the cyclin B-CDK1 complex and G2 arrest, which favors DNA repair [181].

Debromohymenialdisine (DBH), isolated from *Stylissa flabeliformis*, is a potent inhibitor against Chk1 and Chk2, without effect on ATM or ATR in human breast cancer cells with a dominant negative mutant 53-defective. Of note, it exhibited this inhibitory activity at concentrations 8-fold lower than its IC_50_ [182]. DBH used at a non-cytotoxic concentration (3 μM) in association with radiotherapy (2 or 5 Gy) was able to reduce Chk1/2 phosphorylation in human breast cancer cells (MCF7). The combined treatment induced a lower MCF7 survival rate compared to only radiotherapy. The effect was stable over time (24–72 h). Furthermore, DBH reduced the rate of cancer stem cell subpopulation in a time-dependent manner, reaching 65% of inhibition 8 days after irradiation [156]. However, an antagonistic effect was observed in combination with oxaliplatin in human colon cancer cells: cytotoxicity and apoptotic events as well as DNA cross-link formation induced by oxaliplatin were antagonized by DBH [183].

Usually, the strategy adopted in planning combination therapy is to select two drugs with different mechanisms of action, better if they modulate two different pathways. In the recent past, however, the association between compounds acting on the same target achieved resounding success. One example of the latter-mentioned strategy is the association of two microtubule stabilizer agents, i.e., (+)-discodermolide, extracted from *Discodermia dissoluta*, and paclitaxel. This combination therapy led in non-small cell lung cancer cells to a synergistic suppression of microtubule dynamic parameters, such as the shortening rate and length or the length-based rescue frequency (the period of growth or shortening excursions or the period of pause). In the cellular context, these events signified a synergistic increase in G2/M arrest and apoptotic events [158]. The synergism between discodermolide and taxol was also observed in human ovarian carcinoma cells. The antiproliferative effect was synergic at not equimolar ratios and at low concentrations (≤20 nM), which induced aneuploidy and caspase-independent cell death. Only at higher concentrations, was a mitotic arrest observed, as previously reported by Honore et al. [158,159]. Likewise, a combination therapy exploiting low doses of both drugs (discodermolide at 5 mg/kg and taxol at 20 mg/kg) showed a synergistic tumor suppression and antiangiogenic activity in ovarian cancer xenograft-bearing mice, without development of side effects [159]. It is worth highlighting that the ability of taxol to inhibit endothelial cell chemotaxins and invasiveness occurred at concentrations lower than its endothelial antiproliferative activity [184]. Of note, the association between low doses of two stabilizer agents instead of high doses of a single drug most likely means lower treatment toxicity and improvement of therapeutic compliance. The license of discodermolide was bought by Novartis, but the clinical development of the compound was halted because of its toxicity [185]. The research is now focusing on synthetic analogues or hybrid molecules [186].

## 5. Chemoprevention

The high systemic toxicity and the high failure rates of traditional cancer therapies have enhanced the search for new agents, which could prevent and/or slow down cancer growth and, at the same time, improve patients’ compliance [187]. Indeed, carcinogenesis is a long and complex process that presents many opportunities for interventions. For many types of cancer, such as breast malignancies, the WHO specifically suggested chemoprevention as an effective therapy.

Somatic cell mutations are pivotal in tumor initiation and in the other phases of carcinogenesis. By definition, chemopreventive agents trigger protective mechanisms either inside cells or in the extracellular environment in order to prevent or inhibit DNA mutations and cancer initiation. Since 1995, it has been known that marine organisms and, in particular, marine sponges do not develop cancer. This is due to their efficient bio-transformation and detoxification systems that counteract the generation of DNA lesions induced by carcinogens [188,189]. This evidence stimulated the idea that sponges and their metabolites could exert chemopreventive activity in humans. So far, only a few compounds isolated from marine sponges have been tested for their ability to inhibit cancer initiation.

*Arenosclera brasiliensis* ethanolic extract is one example of a potential chemopreventive agent obtained from marine sponges. The crude extract was not genotoxic per se and prevented DNA damage [190]. Indeed, *Salmonella typhimurium* reverse mutation tests (Ames test) or direct plasmid treatments showed that the extract prevented mitomycin C, sodium azide, and 2-aminofluorene mutagenicity [190], by acting through intracellular and extracellular mechanisms and favoring DNA repair.

The antioxidant verongiaquinol and manzamine A, isolated from the red sea sponge *Aplysina* sp. and *Acanthostrongylophora* sp., respectively, are other two antimutagenic compounds. They reduced base-pair substitution, frame-shift and transitional mutagenicity induced by sodium azide and methyl methanesulfonate, thus probably acting through different mechanisms [191]. The proposed mechanisms of action involved the adsorption of mutagens [192,193] or the induction of DNA glycosylase enzymes, which repair alkylated DNA bases [194].

The ethanolic extract of *Haliclona koremella* has antioxidant and free-radical scavenger characteristics, which are responsible for its antimutagenic activity. In fact, the extract reduced the number of micronucleated polychromatic erythrocytes induced by metronidazole, a drug whose genotoxic potential is related to the induction of oxidative stress [195].

## 6. Clinical Studies

It is worth noting the fact that, despite being successful in preclinical models, around 95% of new anticancer drugs fail in clinical trials [196]. Furthermore, many compounds showing very promising properties in in vitro models suffer pharmacokinetic problems in animal models [197].

To date, only one anticancer drug derived from marine sponges is commercially available: eribulin mesylate (brand name Halaven). Eribulin mesylate (EM) is a synthetic analog of helicondrin B, extracted from the *Porifera Halichondria okadai* [198]. It was approved by the FDA in 2010 [199] and by the European Medicines Agency (EMA) in 2011 for the treatment of metastatic breast cancer or locally advanced breast cancer as third-line therapy after two chemotherapeutic regimens with an anthracycline and a taxane [200]. In 2014, the American Society of Clinical Oncology recommended EM as a second- and later-line chemotherapy in women with human epidermal growth factor receptor 2 (HER2)-negative advanced breast cancer [201].

EM is a microtubule destabilizing agent that increases the formation of aberrant mitotic spindles leading to an irreversible mitotic arrest [202]. It exhibited a selective activity for proliferating cells since no effect was recorded in human quiescent immortalized fibroblasts [203]. Differently from what was observed for other microtubule-depolymerizing compounds [204], EM acts on the abnormal tumor vasculature, where it increases the formation of microvessels and, therefore, tumor perfusion [205,206]. The enhanced vascularization could lead to a duplex benefit: the improved delivery of drugs into the hypoxic core of the tumor and the reduction of chemoresistance driven by hypoxia [207]. Another characteristic of EM, which could positively affect the outcome, is to reverse the epithelial-to-mesenchymal transition. During this process, epithelial cells adopt the mesenchymal phenotype, which is related to drug resistance, invasion, metastasis, and changes in stem cell phenotype [208]. EM was able to reverse this transition in three triple-negative breast cancer cell lines, decreasing their mesenchymal gene and protein profiles in favor of the epithelial one [209]. In preclinical studies, EM showed antitumor activity against many types of cancer, such as breast, ovarian, colon, pancreatic, head and neck, non-small-cell and small-cell lung cancer, leiomyosarcoma, fibrosarcoma, glioblastoma, melanoma, and acute lymphocytic leukemia [203,210,211]. In clinical trials performed on patients with locally advanced or metastatic cancer, EM increased the progression-free and overall survival compared to the reference drug, with manageable side effects, such as neutropenia, fatigue, and peripheral neuropathy [212,213].

In 2016, the FDA approved EM for the treatment of metastatic or unresectable liposarcoma as second-line chemotherapy after a prior anthracycline-containing regimen. Moreover, EM was designed as an orphan drug, encouraging its development for future applications [214]. In the phase III clinical trial on previously treated patients with advanced liposarcoma or leiomyosarcoma, EM increased overall survival compared to dacarbazine (13.5 vs. 11.5 months). However, patients developed intermediate or serious adverse effects more frequently than when treated with the reference drug (67% vs. 56%); mortality was higher than dacarbazine (4% vs. 1%) [215]. Adverse effects include neutropenia, numbness and peripheral neuropathy, and cardiomyopathy with risk of death [215].

To date, there are 131 clinical trials on EM, among them 84 regarding breast cancer (53 are ongoing) [216]. In 28 clinical trials (8 have been completed or terminated), EM was studied as a combination therapy with other antineoplastic drugs, such as carboplatin (NCT03032614), gemcitabine (NCT00410553), or irinotecan (NCT02318589), but also with new compounds like PQR309 (NCT02723877) or POL6326 (NCT01837095) [217].

E7974 is a synthetic analog of hemiasterlin, isolated from the marine sponge *Hemiasterella minor* [218]. E7974 inhibits microtubule polymerization binding mostly to the α-subunit into the α/β-tubulin heterodimer interface. It alters the architecture of the mitotic spindles, induces G2/M arrest, and apoptosis [219]. It showed a strong antiproliferative activity against many cancer cell lines [219]. It was also effective in paclitaxel-resistant cell lines and in human xenograft-cancer models sensitive or not to paclitaxel. It does not bind to β-tubulin, the major site of mutations in taxane-resistant cell lines, and is a poor substrate of Pgp efflux pump [220,221]. So far, three clinical phase I studies with different schemes of drug administration have been completed (NCT00165802, NCT00130169, NCT00121732) [217]. They were conducted in patients with refractory solid tumors, and the disease was stabilized in 35%. The most relevant toxicity was neutropenia. However, all toxic effects were reversible and manageable [221].

Up to now, there are two phase I clinical studies on PM060184; one has been completed (NCT01299636) and one is in the recruiting status (NCT02533674) [217]. The completed clinical trial enrolled 20 patients with advanced solid tumor such as breast cancer, non-small cell lung cancer, and urothelial cancer. One patient showed partial response and three patients achieved disease stability. Severe developed toxicities were bowel obstruction, vomiting, tumor pain, peripheral motor neuropathy, neutropenia, and thrombocytopenia [222]. The ongoing clinical study will deal with the association between PM060184 and gemcitabine in patients with advanced solid tumors [217].

Considering the high social impact of cancer pathology, finding new therapeutic strategies for the treatment of late-stage or refractory tumors is essential. Taking into account the fact that such drugs should improve the quality of life of those patients that are already physically and psychologically devastated by the disease and the chemotherapy, the authorization by the FDA for these drugs has been simplified. In particular, for this category of drug products a deferral of genotoxicity studies during the initial phase of clinical drug development is authorized [223]. Usually, these compounds are characterized by a slightly better overall- or progression-free-survival than the reference-listed drug, e.g., EM versus decarbazine. Moreover, although adverse reactions are common, they are reversible and manageable [221]. However, for clinical trials enrolling patients at early stages of cancer such as EM in NCT01439282 and NCT01593020 studies, the exclusion of genotoxic and mutagenic studies should not be recommended. However, genotoxic studies have been reported for only very few natural products originated from sponges. Ingenamine G from *Pachychalina alcaloidifera* proved genotoxic in human proliferating lymphocytes [224].

## 7. Conclusions

**Figure 1:**
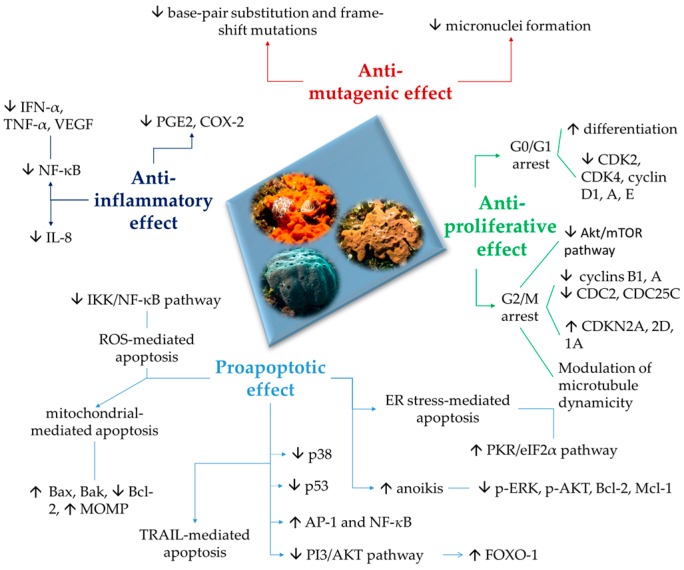
Pathways involved in anti-mutagenic, anti-inflammatory, anti-proliferative and proapoptotic effects of the marine compounds.

The increasing knowledge of the ability of compounds isolated from marine sponges to target various events of the carcinogenetic process suggests that they could serve as new tools for both preventive and therapeutic interventions. The evidence reported in this review highlights the great value of marine sponge derivatives as a promising source of anticancer compounds. The compounds presented here evoked their potential antitumor activity by different mechanisms, such as cell-cycle arrest, anti-inflammatory activity, apoptosis, induction of ER stress, and interaction with many different targets involved in cancer development, such as mitochondrial membrane, PARP, cytochrome *c*, Akt, and caspases (Figure 1).

More than one pathway is usually affected by each sponge derivative. Aaptamines, for example, are capable of triggering apoptosis, inhibiting cell proliferation and acting as cytoprotective elements against traditional chemotherapeutic agents. This highlights their multi-target nature enabling them to effectively counteract the biological complexity of cancer. One aspect worthy of note is that some marine sponge derivatives exhibit potential anticancer effects through particular and partially novel mechanisms. An example is eribulin, which modulates microtubule dynamics through a mechanism different from other antimicrotubular chemotherapy drugs. 

Moreover, many sponge compounds showed additive or synergistic effects when tested with traditional antiblastic drugs. This strategy is very interesting since it makes it possible to lower the dose of traditional antitumor drugs, thus decreasing their toxicity and improving their therapeutic index. In addition, the combined therapy can circumvent the insurgence or overcome multidrug resistance, which still represents a significant limitation for traditional anticancer treatments. The synergy they show indicates these molecules as very interesting leads for drug development. Thus, future research should focus on the assessment and exploitation of the clinical anticancer potential of the most promising candidates isolated from marine sponges, balancing both their broad-spectrum effectiveness and their toxicity in order to carefully define their risk–benefit ratio.

## Figures and Tables

**Table 1:** Apoptosis-inducing compounds isolated from marine sponges.

Compounds	Sponge	Cell Line	Concentration Range (μM ^a^)	Chromatin Condensation/DNA Fragmentation	Phosphatidylserin Externalization	Caspase Activation	PARP Cleavage	Reference
Aaptamine	*Aaptos* sp.	THP-1	50–200		√			[14]
Demethyl(oxy)aaptamine	*Aaptos* sp.	THP-1	10–25		√			[14]
Isoaaptamine	*Aaptos* sp.	THP-1	10–25		√			[14]
Microsclerodermin A	*Amphibleptula* sp.	AsPC-1	2.4	√		3 and 7		[15]
		BxPC-3	2.4	√		3 and 7		
		PANC-1	2.4	√		3 and 7		
Isofistularin-3	*Aplysina aerophoba*	Raji	50	√		3 and 7	√	[16]
		U937	50	√		3 and 7	√	
Spongiatriol	*Australian spongia* sp.	AsPC-1	6.8	√		3 and 7		[17]
		PANC-1	6.8	√		3 and 7		
		MIA PaCa-2	6.8			3 and 7		
		BxPC-3	6.8			3 and 7		
Laulimalide	*Cacospongia mycofijiensis*	MDA-MB-435	0.1			3	√	[18]
Scalaradial	*Cacospongia scalaris*	HeLa	10 μg/mL			3		[19]
		T47D	10 μg/mL	√				
Candidaspongiolide	*Candidaspongia* sp.	U251 HCT116	0.05–0.10		√	3 and 12	√	[20]
Callyspongidiol	*Callyspongia* sp.	HL-60	31.0–77.5	√				[21]
Crambescidin 800	*Crambe crambe*	HepG2	0.5–2.5		√	3		[22]
Crambescidin 816	*Crambe crambe*	HepG2	0.5–2.5		√	3		[22]
Crambescidin 830	*Crambe crambe*	HepG2	0.5–2.5		√	3		[22]
Smenospongine	*Dactylospongia elegans*	U937	5–15	√				[23]
		HL-60						
Pectenotoxin-2	*Dinophysis fortii* and *Dinophysis acuminata*	Hep3B	0.01 μg/mL		√	3, 8 and 9		[24]
		U937	0.008–0.010 μg/mL	√	√	3	√	[25,26]
Salarin C	*Fascaplysinopsis* sp.	K562 normoxic and hypoxic conditions	0.01–0.2	√	√	3 and 9	√	[27,28]
Cacospongionolide	*Fasciospongia cavernosa*	Hela	10 μg/mL			3		[19]
		T47D	10 μg/mL	√				
Lasonolide A	*Forcepia* sp.	CA46, Ramos, Daudi, HL-60, MDA-MD-231, MCF-7, HCT-116, HT-29	0.1	√				[29]
Geoditin A	*Geodia japonica*	HL-60	1.6 to 25 μg/mL	√	√	3		[30,31]
		HT-29	5–30	√				
Stellettin A	*Geodia japonica*	HL-60	4 μg/mL			3		[32]
Ilimaquinone	*Hippospongia metachromia*	HCT116	2.5–10	√	√	3 and 8	√	[33,34]
		PC-3	2–10	√				[35]
Spongistatin 1	*Spirastrella spinispirulifera,* *Hyrtios erecta*	MCF-7	0.0002–0.0005	√		slight activation of 2, 3, 6, 7, 8 and 9		[36]
		Jurkat	0.0002	√		2, 3, 7, 8 and 9	√	[37]
		L3.6pl	0.00001–0.01	√				[38]
Heteronemin	*Hyrtios* sp.	K562	1.4–5.6	√	√	3, 8 and 9	√	[39]
		DU145	0.01–1 μg/mL	√		3, 8 and 9		[40]
		PC-3	0.01–1 μg/mL	√		3, 8 and 9		
		LNCaP	0.01 μg/mL	√				
		T24	0.1–0.8 μg/mL	√	√	3 and 9	√	[41]
		A498	0.5–3	√		3, 8 and 9	√	[42]
Bastadin 6	*lanthella* sp.	HUVEC	0.01–1	√		3 and 7		[43]
Irciniastatin A	*Ircinia ramose, Psammocinia* sp.	Jurkat	0.01	√		3, 8 and 9		[44]
Jaspolide B	*Jaspis* sp.	Bel-7402 HepG2	0.5 10–20	√ √				[45]
Stellettin B	*Jaspis stellifera*	K562	0.012–0.054		√	3 and 9	√	[46]
		A549	0.02–1		√	3 and 7	√	[47]
		SF295	0.04–1		√		√	[48]
Jaspine B or Pachastrissamine	*Jaspis* sp. *Pachastrissa* sp.	B16 HaCaT	5 5 μg/mL	√	√	3 and 9 3	√	[49,50]
Petrosterol-3,6-dione	*Lanthella* sp.	HL-60	19.9	√				[51]
5α,6α-epoxy-petrosterol	*Lanthella* sp.	HL-60	21.3	√				[51]
petrosterol	*Lanthella* sp.	HL-60	21.5	√				[51]
Leiodermatolide	*Leiodermatium* sp.	AsPC-1	0.01	√				[52]
		BxPC-3	0.01	√		3		
		MIA PaCa-2	0.01	√		3		
Naamidine A	*Leucetta chagosensis*				√	3, 8 and 9	√	[53]
Monanchocidin A	*Monanchora pulchra*	HeLa	1.39 2.01	√	√	3 and 7		[54]
Monanchocidin B	*Monanchora pulchra*	HeLa	0.58 1.36	√		3 and 7		[54]
Monanchocidin C	*Monanchora pulchra*	HeLa	1.84 1.31	√		3 and 7		[54]
Ptilomycalin A	*Monanchora pulchra*	HeLa	1.1 0.5	√		3 and 7		[54]
Monanchomycalin B	*Monanchora pulchra*	HeLa	1.5 1.72	√		3 and 7		[54]
Normonanchocidin D	*Monanchora pulchra*	HeLa	2.1 5.2	√		3 and 7		[54]
Urupocidin A	*Monanchora pulchra*	HeLa	28.7 27	√				[54]
Pulchranin A	*Monanchora pulchra*	HeLa	51 58	√		3 and 7		[54]
Pateamine	*Mycale* sp.	32D	0.1		√			[55]
Mycalamide A	*Mycale* sp.	32D	0.1		√			[55]
Latrunculin A	*Negombata magnifica*	MKN45 NUGC-4	−10 0.01–10			3 and 7		[56]
Kuanoniamines A	*Oceanapia sagittaria*	MCF-7	0.5–2.5	√				[57]
Kuanoniamines C	*Oceanapia sagittaria*	MCF-7	1.0–2.5	√				[57]
Dideoxypetrosynol A	*Petrosia* sp.	SK-MEL-2	0.1–0.3 μg/mL	√		3 and 9	√	[58]
Psammaplin A	*Psammaplysilla* sp.	Human endometrial Ishikawa		√				[59]
Psammaplysene A	*Psammaplysilla*	Ishikawa ECC1	1 1				√	[60]
(1′*R*,5′*S*,6′*S*)-2-(3′,5′-dibromo-1′,6′-dihydroxy-4′-oxocyclohex-2′-enyl) acetonitrile	*Pseudoceratina* sp.	K562	7.7–30.8		√	3 and 9	√	[61]
13*E*,17*E*-globostellatic acid X methyl ester	*Rhabdastrella globostellata*	HUVEC	1–10			3 and 7		[51]
Rhabdastrellic acid-A	*Rhabdastrella globostellata*	HL-60		√		3	√	[62]
Rhizochalin or Rhizocalinin	*Rhizochalina incrustata*	HL-60	10–25		√	3, 8 and 9		[63]
		HT-29	1–6	√	√	3	√	[64]
		THP-1	1–10		√		√	[65]
		PC-3	0.5–4		√	8	√	[66]
		DU-145	0.5–4		√	8	√	
		22Rv1	0.5–4		√	8	√	
		VCaP	0.5–4		√	8	√	
Ircinin-1	*Sarcotragus*	SK-MEL-2	25–50	√		3 and 9	√	[67]
Sipholenol A	*Siphonochalina* sp.	HepG2	17.18	√		3		[68]
		HCT-116	14.8	√		3		[69]
Sipholenol L	*Siphonochalina* sp.	HepG2	24	√		3		[68]
		HCT-116	19.8	√		3		[69]
Smenamides A and B	*Smenospongia aurea*	Calu-1	0.05–0.1		√			[70]
(Z)-stellettic acid C	*Stelletta* sp.	U937	17.2–103.3		√	3, 8 and 9		[71]
Renieramycin M	*Xestospongia* sp.	H460	5–40	√				[72,73]
		U373MG	0.0031			3	√	

^a^ If not specified.

**Table 2:** Antiproliferative compounds isolated from marine sponges.

Compounds	Sponges	Cell Lines	Concentrations (μM ^a^)	Phase of Cell-Cycle Arrest	Molecular Targets	Reference
Aaptamine	*Aaptos aaptos*	NT2	1–50	G2/M	/	[106]
		HepG2	50–100	G2/M	↓ cyclins D and E, CDK2 ↑ p21	[107]
		HCC-LM3		S		
	*Aaptos suberitoides*	MG63	30 μg/mL	G2/M	↑ p21	[108]
		K562	20–100	G2/M	↑ p21	[109]
Aphrocallistin	*Aphrocallistes beatrix beatrix*	Panc-1	≤46.5	G0/G1	/	[110]
Aragusterol A	*Xestospongia* sp.	A549	1–10	Late G1	↓ CDK2, CDK4 ↓ cyclins D1, A, E ↓ pRb	[111]
Batzelline A and B Isobatzelline A, C, D Secobatzelline A and B	*Batzella* sp.	AsPC-1	5 or 25 μg/mL	S	Intercalate into DNA and/or inhibit Topoisomerase II activity	[112]
Isobatzelline E				G2/M		
Calyculin A	*Discodermia calyx*	MDA-MB-468 MCF-7 MDA-MB-231	0.01	G0/G1	↓ cyclin D1	[113]
Crambescidin 800	*Monanchora ungiculata*	K562	0.15–1.5	S	↑ p21	[114]
Crambescidin 800, 816 and 830	*Crambe crambe*	HepG2	2.5	G0/G1	↓ cyclins A, D ↓ CDK2, 6, 1 ↑ CDKN2A, 2D, 1A	[22]
Dictyostatin-1	*Corallistidae* sp.	A549	0.01–1	G2/M	↑ micronuclei, asters and abnormal mitotic spindles formation	[115]
Dideoxypetrosynol A	*Petrosia* sp.	U937	0.2–1 μg/mL	G0/G1	↑ cyclin D1 ↓ cyclin E ↑ pRB-E2F1 complex and p16	[116]
(+)-Discodermolide	*Discodermia dissoluta*	MCF-7, CA46	0.01–1	G2/M	Stabilize microtubules	[117]
		A549	0.07–0.166	G2/M	abnormal mitotic spindles ↓ microtubules dynamicity	[118]
Geodiamolide A, B, H and I	*Geodia corticostylifera*	T47D, MCF7	50 ng/mL	Not investigated	↑ disorganization of actin filaments	[119]
(19*Z*)-Halichondramide	*Chondrosia corticata*	A549	0.025–0.1	G2/M	↑ p53, GADD45 ↓ CDC2, CDC25C, cyclin B1, cyclin A	[120]
Hemiasterlin, Hemiasterlin A and B	*Hemiasterella minor*	MCF-7	0.0005–0.01	G2/M	↑ abnormal mitotic spindles formation	[121]
Jaspolide B	*Jaspis* sp.	Bel-7402 HepG2	20	G0/G1	↑ microtubule disassembly	[45]
Laulimalide	*Cacospongia mycofijiensis*	MDA-MB-435	0.02	G2/M	Microtubule stabilization	[18]
		A-10 SK-OV-3	0.02–2		↑ micronuclei and abnormal mitotic spindles formation	
Leiodermatolide	*Leiodermatium* sp.	PANC-1	0.01–0.1	G2/M	↓ mitotic spindles formation and microtubule elongation	[52]
		A549	0.01–1	G2/M	↓ mitotic spindles formation	[122]
		U2OS	0.018–0.23	G2/M	Tubulin disruption Centrosome amplification Micronuclei formation	[123]
Pachymatismin	*Pachymatisma johnstonii*	DU145	4–16		Microtubules depolymerization	[124]
		NSCLC-N6	2–20 μg/mL	G0/G1		[125]
		NSCLC-N6 subcutaneous xenografts	0.5–5 mg/kg		↓ tumor growth	[126]
Peloruside A	*Mycale hentscheli*	H441	0.01–1	G2/M	Microtubule stabilization, ↑ micronuclei and abnormal mitotic spindles formation	[127]
		MCF-7	0.025–0.1	G2/M	↓ microtubule dynamicity (growth rate, growth length, time spent growing)	[128]
PHC-1	*Phyllospongia chondrodes*	K562	0.1–5 μg/mL	G0/G1	↑ haemoglobin, glycophorin A and enucleation	[129]
PM050489, PM060184	*Lithoplocamia lithistoides*	A549	0.25–1 × 10^−3^	G2/M [130]	↓ microtubules formation binding αβ tubulin dimers	[131]
PM060184			0.001		↑ abnormal mitotic spindles formation, ↓ CDK1, ↑ p21	[132]
		HCT116	0.01		↑ formation of multinucleated cells	
		MDA-MB-231 subcutaneous xenografts	16 mg/kg			
Sipholenol-A	*Siphonochalina siphonella*	PC-3	7.9	G0/G1	/	[133]
Smenospongine	*Dactylospongia elegans*	K562	3–15	G0/G1	↑ p21, ↓ p57, ↓ pRb; ↑ haemoglobin, glycophorin A	[23,134]
(8*E*,13*Z*,20*Z*)-strobilinin/(7*E*,13*Z*,20*Z*)-felixinin 1:1	*Psammocinia* sp.	HeLa	10–50	S	↓ topoisomerase I and polymerase alpha-primase activities	[130]

^a^ If not specified.

**Table 3:** Biological effects of compounds isolated from marine sponges in association with radiotherapy or anticancer chemotherapy.

Drug Associations	Sponge	Cell Line	Concentrations (μM ^a^)	CI	Biological Effect	Reference
Debromohymenialdisine (DBH) + Radiotherapy	*Stylissa flabeliformis*	MCF-7	3 (DBH) + 2–5 Gy		↓ pChk1/2, survival rate and cancer stem cell subpopulation	[156]
(+)-Discodermolide (D) + Taxol (T)	*Discodermia dissoluta*	A549	0.1–5 (T) + 0.5–25 (D) (1:5 molar ratio)	0.396 ^b^	↑ antiproliferative effect and aneuploidy	[157]
		MCF-7		0.273 ^b^		
		SKOV-3		0.476 ^b^		
(+)-Discodermolide + Paclitaxel (PT)		A549	0.07 (D) + 0.02 (PT)	0.59 ± 0.04	Microtubules stabilization G2/M arrest apoptosis	[158]
(+)-Discodermolide + Taxol		SKOV-3	0.001 (D) + 0.02 (T) or 0.02 (D) + 0.001 (T)	≤0.7	↑ antiproliferative effect and aneuploidy	[159]
		SKOV-3 xenograft-bearing athymic (*nu*/*nu*) female mice	5 mg/kg (D) + 20 mg/kg (T)		↓ tumor volume and vascularization	
Fascaplysin (F) + Camptothecin (C) 10-hydroxycamptothecin (HC)	*Fascaplysinopsis* Bergquist sp.	NCI-H417	0.5 (F) + 0.5 (C)	0.53		[160]
			1 (F) + 2 (HC)	0.82		
Panicein A (PA) + Doxorubicin (Doxo)	*Haliclona (Soestella) mucosa*	MEWO	10 (PA) + 2 (Doxo)		↓ IC_50_ and Doxorubicin cellular efflux ↑ apoptosis	[161]
		A375	25 (PA) + 1.5 (Doxo)			
Peloruside A (P) + Paclitaxel (PT)	*Mycale hentscheli*	1A9	0.005–0.02 (P)+ 0.005–0.015 (PT)	0.48–0.96	↑ G2/M arrest	[162]
		HL-60	0.015–0.03 (P) + 0.02–0.04 (PT)	0.16–0.87		
Peloruside A (P) + Epothilone A (E)		1A9	0.005–0.025 (P) + 0.005–0.01 (E)	0.41–0.96	↑ G2/M arrest	
		HL-60	0.02–0.125 (P) + 0.01–0.02 (E)	0.08–1.04		

CI = combination index. CI ≤ 0.7: synergy; 0.7 < CI < 1.2: additivity; CI ≥ 1.2 antagonism; ^a^ If not specified; ^b^ CI as a mean.
